# Supplementary material for: Immunization with Herpes Simplex Virus Nanoparticles Targeting Both Attachment and Fusion Protect Against Infection
Source: bioRxiv. 2026 Apr 29:2026.04.24.720674. Preprint. [Version 2] doi: 10.64898/2026.04.24.720674 (PMC13182188; doi:10.64898/2026.04.24.720674)

bioRxiv preprint doi: https://doi.org/10.64898/2026.04.24.720674; this version posted April 29, 2026. The copyright holder for this preprint (which was not certified by peer review) is the author/funder. This article is a US Government work. It is not subject to copyright under 17 USC 105 and is also made available for use under a CC0 license.

Supplemental Figures

Supplemental Figure 1. Purification of vaccine components and negative stain electron microscopy of nanoparticles. Refers to Figure 1.

(A) Diagram of SpyCatcher003-mi3 NP construct.

(B) SDS-PAGE with Coomassie staining of NP fractions after size exclusion chromatography purification (L-Ladder, F-Fraction). Gels converted to Coomassie blue color in PowerPoint.

(C) SDS-PAGE with Coomassie staining of gel for products of SpySwitch purification of soluble gD (L-Ladder, SNT-Supernatant, FT-Flowthrough, W-Wash, E-Elution). Gels converted to Coomassie blue color in PowerPoint.

bioRxiv preprint doi: https://doi.org/10.64898/2026.04.24.720674; this version posted April 29, 2026. The copyright holder for this preprint (which was not certified by peer review) is the author/funder. This article is a US Government work. It is not subject to copyright under 17 USC 105 and is also made available for use under a CC0 license.

(D) SDS-PAGE with Coomassie staining of gel for products of SpySwitch purification of soluble gH/gL (L-Ladder, SNT-Supernatant, FT-Flowthrough, W-Wash, E-Elution). Gels converted to Coomassie blue color in PowerPoint.

(E) Negative stain electron microscopy micrographs of NP, gD NP, and gH/gL NP. Red boxes indicate insets shown in Figure 1D.

Supplemental Figure 2. Full length gels presented in the paper. Refers to Figures 1 and 2.

(A) Uncropped SDS-PAGE with Coomassie staining of antigen-NP coupling from Figure 1C (left panel is gD NP, right panel is gH/gL NP). Gels converted to Coomassie blue color in PowerPoint.

(B) HSV-2 titers from vaginal swabs of individual mice on day 5 associated with Figure 2. Bars indicate means and error bars indicate standard deviations. L.O.D. is the limit of detection. *** for p<0.0001.

Supplemental Figure 3. Purification of vaccine components and negative stain electron microscopy of nanoparticles. Refers to Figure 1.

(A) Size exclusion chromatography (SEC) of NPs.

(B) SEC of gD NP. The first peak is gD NP, and the second peak is the excess soluble gD.

(C) SEC of gH/gL NP. The first peak is gH/gL NP, and the second peak is the excess soluble gH/gL.

bioRxiv preprint doi: https://doi.org/10.64898/2026.04.24.720674; this version posted April 29, 2026. The copyright holder for this preprint (which was not certified by peer review) is the author/funder. This article is a US Government work. It is not subject to copyright under 17 USC 105 and is also made available for use under a CC0 license.

(D) Dynamic light scattering of uncoupled NP, gD NP, and gH/gL NP. Numbers represent hydrodynamic average radius with average polydispersion.

Supplemental Figure 4. Cryogenic electron microscopy (CryoEM) of gH/gL NP. Refers to Figure 1.

(A) Exemplary raw micrograph of gH/gL NPs.

(B) Representative 2D classes of gH/gL NPs.

(C) Fourier Shell Correlation (FSC) curves estimating the resolution of the reconstructed gH/gL NP 3D structure.

(D) Viewing direction distribution plot of 3D structure.

(E) CryoEM map (grey) at 10 σ level with PDB 7B3Y (blue) docked in the map.

(F) CryoEM map (grey) at 0.8 σ level shows protrusions from NP subunits.

Supplemental Figure 5. Protection of mice immunized with gD-NP, or gH/gL-NP, or combinations of gD-NP and gH/gL-NP at different ratios from HSV-2 vaginal challenge. Refers to Figures 2 and 3.

BALB/c mice (n=5 per group) were immunized IM with 2 doses of constructs (5 µg) in SAS adjuvant, 3 weeks apart. Mice were intravaginally challenged with 64,000 PFU of HSV-2 strain 333.

(A) Schematic of the study. Sera was analyzed from mice bled 1.5 weeks after the second dose of vaccine.

bioRxiv preprint doi: https://doi.org/10.64898/2026.04.24.720674; this version posted April 29, 2026. The copyright holder for this preprint (which was not certified by peer review) is the author/funder. This article is a US Government work. It is not subject to copyright under 17 USC 105 and is also made available for use under a CC0 license.

(B) Neutralizing antibody titers in serum from vaccinated mice after dose 2. IC_{50} is the dilution of sera that inhibits infection by 50%. Bars indicate means and error bars indicate standard deviations. **p<0.0014, ***p<0.0001.

(C) Mice were monitored daily for clinical signs of HSV-2 infection including vaginal erythema, genital lesions, genital hair loss, ruffled fur, lethargy, abnormal gait, hunched back, or hind-limb paralysis and given a score of 0 (no disease) to 6 (dead) (see Methods). Data points indicate means and error bars indicate standard deviations. There were no statistical differences between groups receiving NPs.

(D) HSV-2 titers from vaginal swabs taken on days 1, 2, 3, 5, and 7 post-challenge. Data points indicate means and error bars indicate standard deviations.

(E) Area under the curve (AUC) analysis of panel D showing 7 day shedding. Bars indicate means and error bars indicate standard deviations. *p<0.0071.

Supplemental Figure 6. Schematic of immune focusing on receptor binding domains of HSV-2 gD enabled by NP display. Refers to Figure 6.

The epitopes[27] of monoclonal antibodies used for the sera competition assay in Figure 6 were mapped onto PDB (2C36) of HSV-1 gD (HSV-1 gD has more of its structure resolved than HSV-2 gD and shares significant amino acid homology). The carboxyl terminus of gD is less accessible since it is oriented towards the NP and epitopes near the amino terminus are freely exposed. This enforced orientation of gD may enable immune focusing on neutralizing epitopes and sterically hinder immune responses to non-neutralizing epitopes.

bioRxiv preprint doi: https://doi.org/10.64898/2026.04.24.720674; this version posted April 29, 2026. The copyright holder for this preprint (which was not certified by peer review) is the author/funder. This article is a US Government work. It is not subject to copyright under 17 USC 105 and is also made available for use under a CC0 license.

Supplemental Figure 1

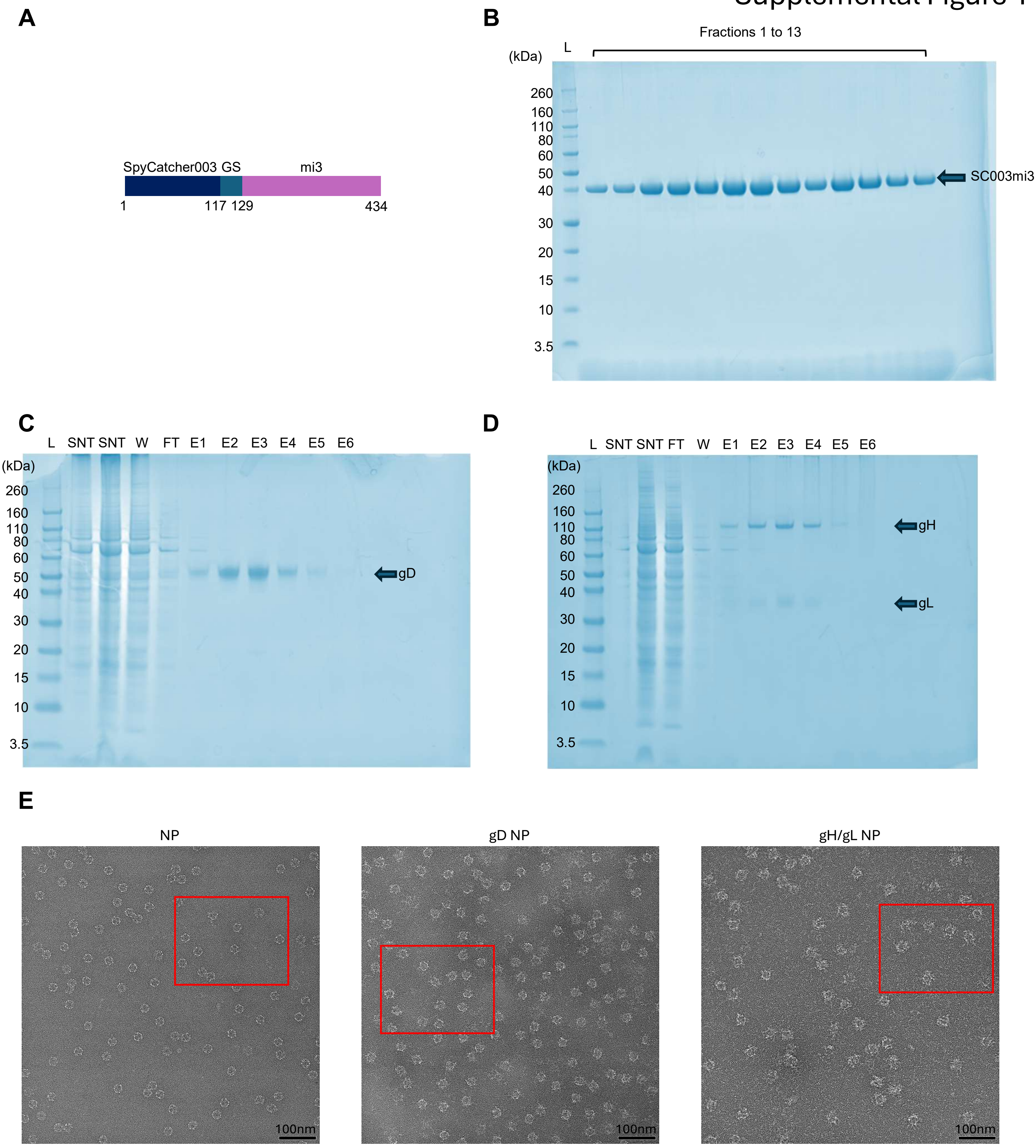

bioRxiv preprint doi: https://doi.org/10.64898/2026.04.24.720674; this version posted April 29, 2026. The copyright holder for this preprint (which was not certified by peer review) is the author/funder. This article is a US Government work. It is not subject to copyright under 17 USC 105 and is also made available for use under a CC0 license.

Supplemental Figure 2

A

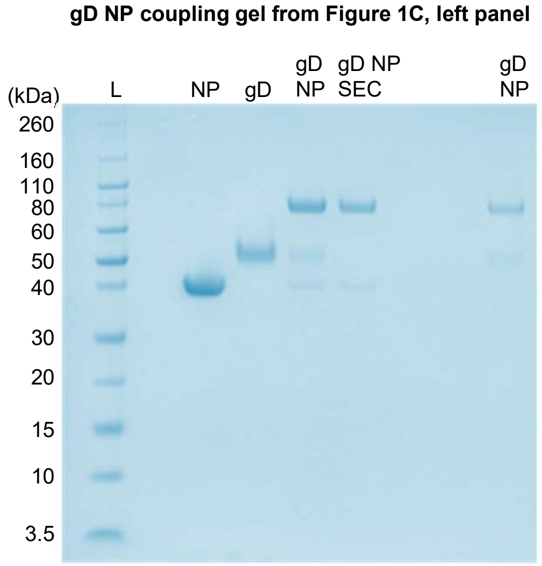

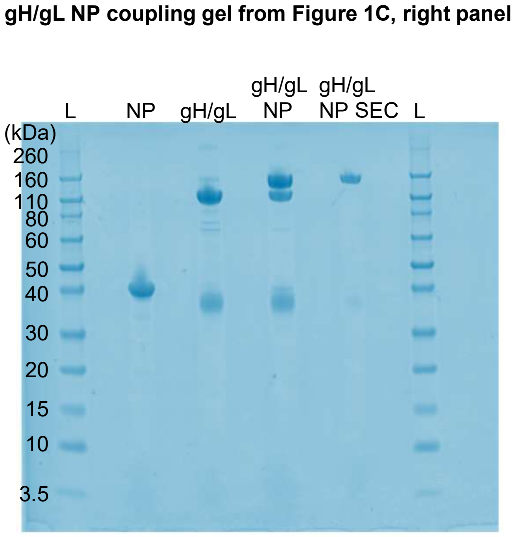

B

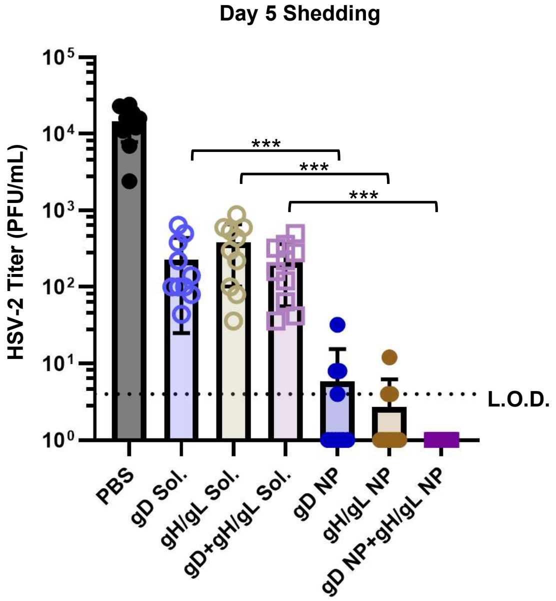

bioRxiv preprint doi: https://doi.org/10.64898/2026.04.24.720674; this version posted April 29, 2026. The copyright holder for this preprint (which was not certified by peer review) is the author/funder. This article is a US Government work. It is not subject to copyright under 17 USC 105 and is also made available for use under a CC0 license.

Supplemental Figure 3

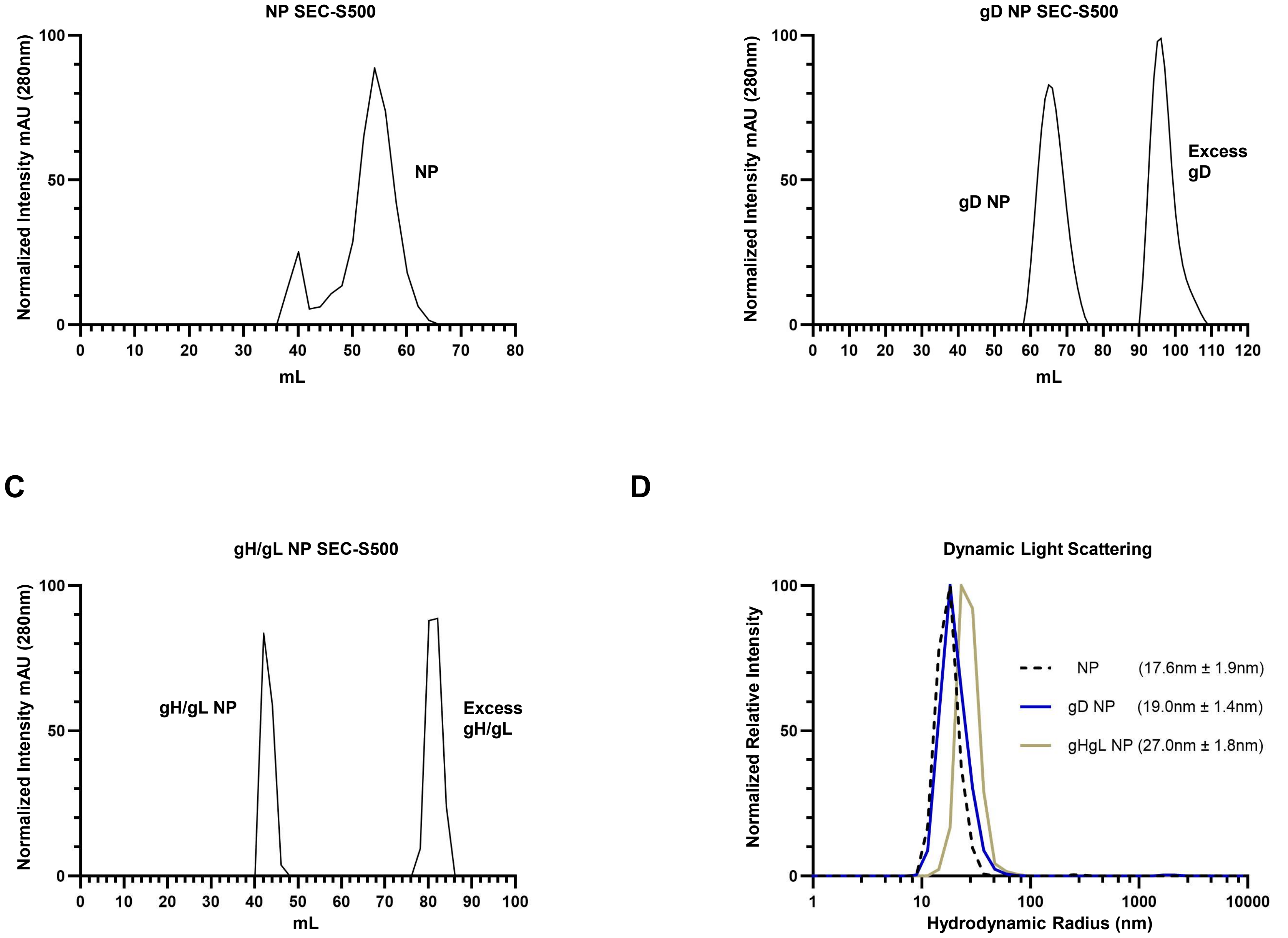

bioRxiv preprint doi: https://doi.org/10.64898/2026.04.24.720674; this version posted April 29, 2026. The copyright holder for this preprint (which was not certified by peer review) is the author/funder. This article is a US Government work. It is not subject to copyright under 17 USC 105 and is also made available for use under a CC0 license.

Supplemental Figure 4

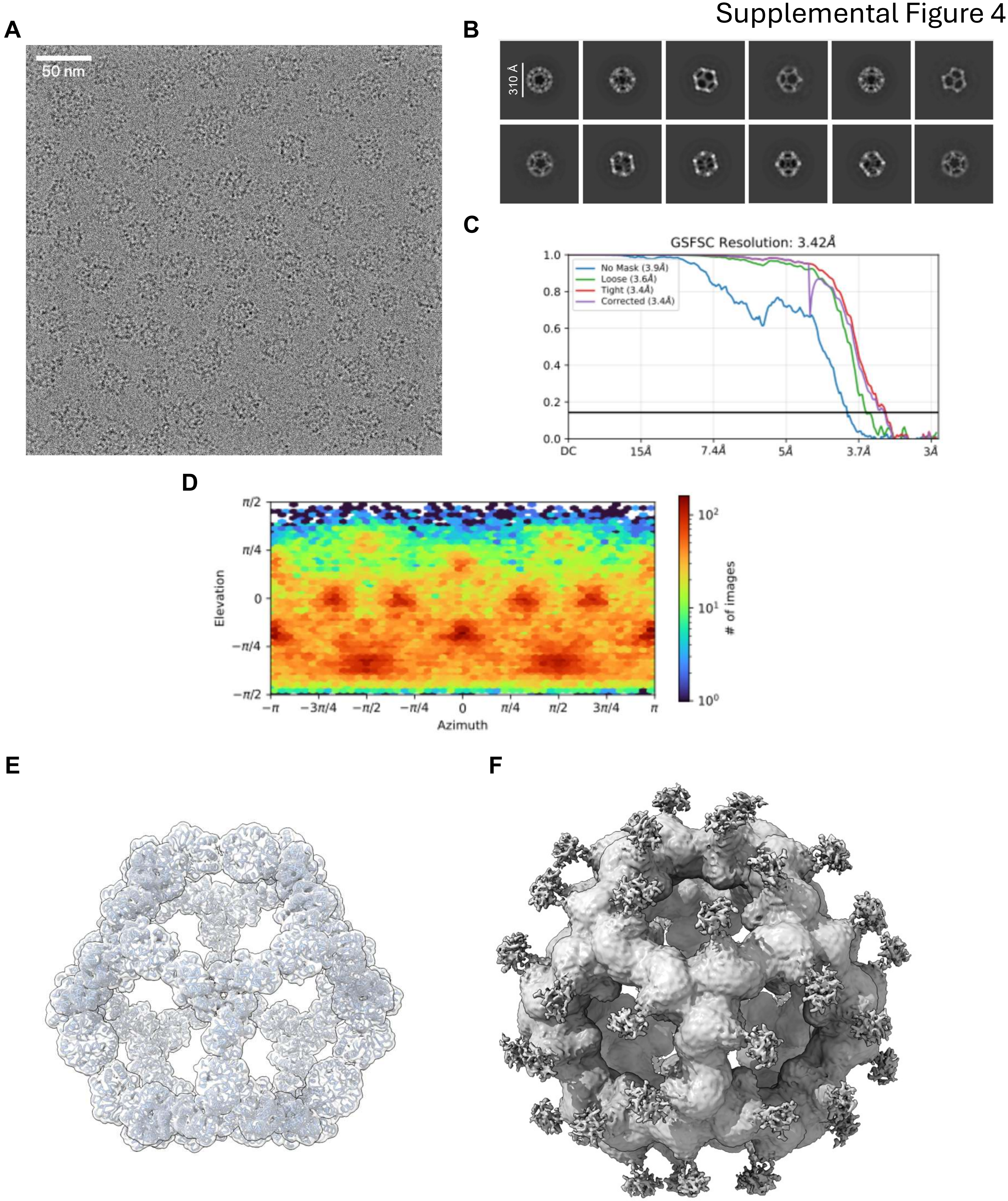

bioRxiv preprint doi: https://doi.org/10.64898/2026.04.24.720674; this version posted April 29, 2026. The copyright holder for this preprint (which was not certified by peer review) is the author/funder. This article is a US Government work. It is not subject to copyright under 17 USC 105 and is also made available for use under a CC0 license.

Supplemental Figure 5

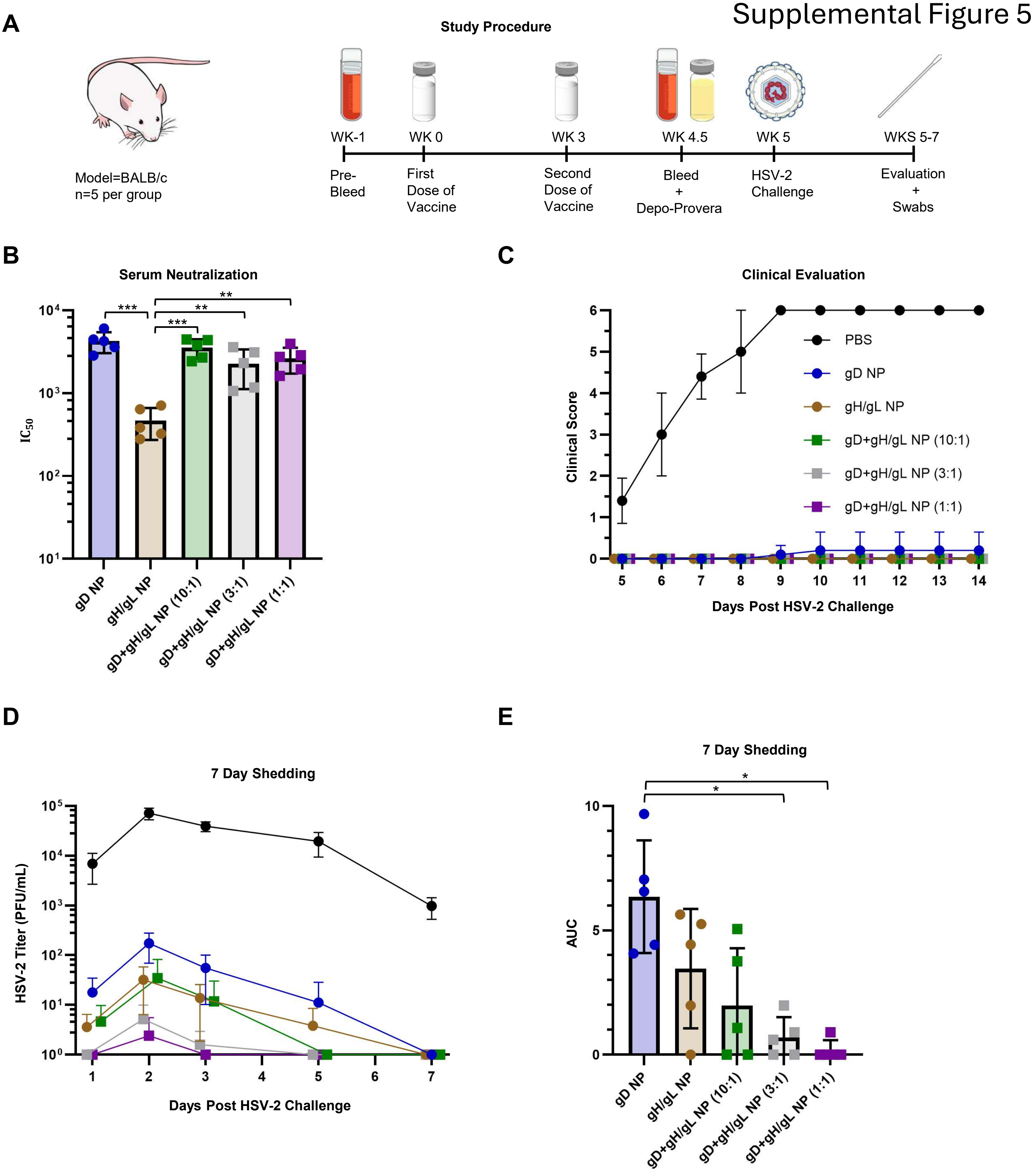

bioRxiv preprint doi: https://doi.org/10.64898/2026.04.24.720674; this version posted April 29, 2026. The copyright holder for this preprint (which was not certified by peer review) is the author/funder. This article is a US Government work. It is not subject to copyright under 17 USC 105 and is also made available for use under a CC0 license.

Supplemental Figure 6

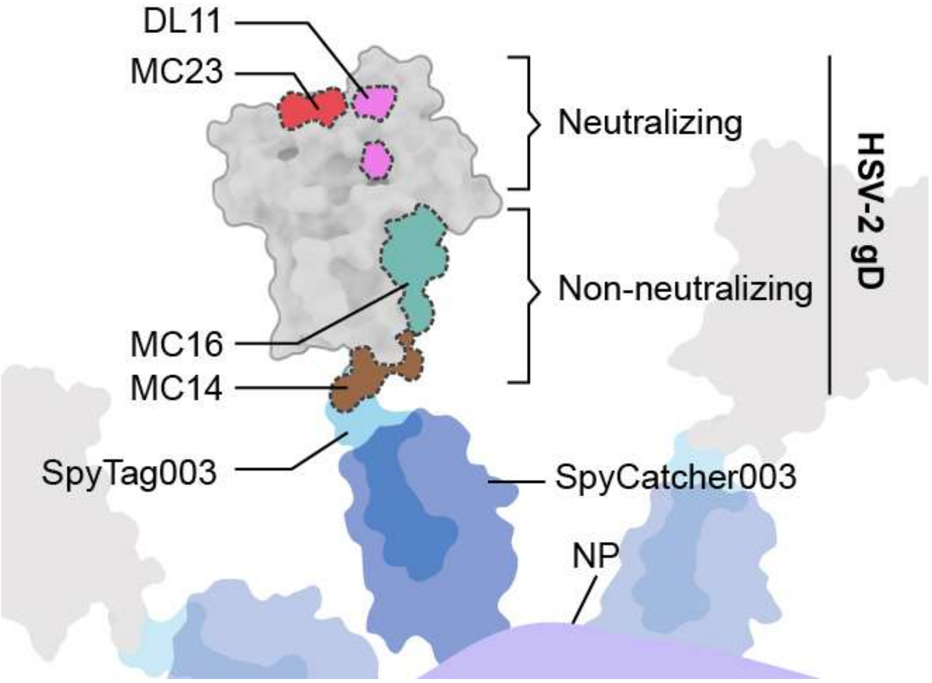

Supplement: 1 [file NIHPP2026.04.24.720674v2-supplement-1.pdf]
